# What to Build for Middle-Agers to Come? Attractive and Necessary Functions of Exercise-Promotion Mobile Phone Apps: A Cross-Sectional Study

**DOI:** 10.2196/mhealth.6233

**Published:** 2017-05-25

**Authors:** Gen-Yih Liao, Yu-Tai Chien, Yu-Jen Chen, Hsiao-Fang Hsiung, Hsiao-Jung Chen, Meng-Hua Hsieh, Wen-Jie Wu

**Affiliations:** ^1^ Department of Information Management Chang Gung University Taoyuan City Taiwan; ^2^ Dept of Nursing Chang Gung Memorial Hospital, Taoyuan Branch Taoyuan City Taiwan; ^3^ Department of Information Management National Taiwan University Taipei Taiwan; ^4^ Department of Nursing Chang Gung University of Science of Technology Guishan District, Taoyuan City Taiwan; ^5^ Division of Chinese Gynecology, Center for Traditional Chinese Medicine Chang Gung Memorial Hospital, Taoyuan, Taiwan Kwei-Shan Tao-Yuan Taiwan

**Keywords:** physical exercise, middle aged, mobile application, self efficacy, consumer preference

## Abstract

**Background:**

Physical activity is important for middle-agers to maintain health both in middle age and in old age. Although thousands of exercise-promotion mobile phone apps are available for download, current literature offers little understanding regarding which design features can enhance middle-aged adults’ quality perception toward exercise-promotion apps and which factor may influence such perception.

**Objectives:**

The aims of this study were to understand (1) which design features of exercise-promotion apps can enhance quality perception of middle-agers, (2) whether their needs are matched by current functions offered in app stores, and (3) whether physical activity (PA) and mobile phone self-efficacy (MPSE) influence quality perception.

**Methods:**

A total of 105 middle-agers participated and filled out three scales: the International Physical Activity Questionnaire (IPAQ), the MPSE scale, and the need for design features questionnaire. The design features were developed based on the Coventry, Aberdeen, and London—Refined (CALO-RE) taxonomy. Following the Kano quality model, the need for design features questionnaire asked participants to classify design features into five categories: attractive, one-dimensional, must-be, indifferent, and reverse. The quality categorization was conducted based on a voting approach and the categorization results were compared with the findings of a prevalence study to realize whether needs match current availability. In total, 52 multinomial logistic regression models were analyzed to evaluate the effects of PA level and MPSE on quality perception of design features.

**Results:**

The Kano analysis on the total sample revealed that visual demonstration of exercise instructions is the only attractive design feature, whereas the other 51 design features were perceived with indifference. Although examining quality perception by PA level, 21 features are recommended to low level, 6 features to medium level, but none to high-level PA. In contrast, high-level MPSE is recommended with 14 design features, medium level with 6 features, whereas low-level participants are recommended with 1 feature. The analysis suggests that the implementation of demanded features could be low, as the average prevalence of demanded design features is 20% (4.3/21). Surprisingly, social comparison and social support, most implemented features in current apps, were categorized into the *indifferent* category. The magnitude of effect is larger for MPSE because it effects quality perception of more design features than PA. Delving into the 52 regression models revealed that high MPSE more likely induces *attractive* or *one*- *dimensional* categorization, suggesting the importance of technological self-efficacy on eHealth care promotion.

**Conclusions:**

This study is the first to propose middle-agers’ needs in relation to mobile phone exercise-promotion. In addition to the tailor-made recommendations, suggestions are offered to app designers to enhance the performance of persuasive features. An interesting finding on change of quality perception attributed to MPSE is proposed as future research.

## Introduction

### Background

Middle age begins when young adulthood ends and ends when old age starts [1]. The United Nation reported that the middle-age population in the more developed regions had increased dramatically from 2000 to 2010 and remain the greatest age group by 2024 [2], suggesting the importance of middle age studies. Because physiological functions (eg, lung function, muscle mass, renal blood flow, and bone density) begin to deteriorate in middle age, scientiﬁc guidelines suggest that physical exercise be taken to counter this deterioration effect [3]. Two studies examined the employees, aged 40-60 years, who changed their leisure time PA and found that increased PA reduced subsequent sickness absence and risk of disability retirement [4,5]. In addition, an empirical study has conducted a 14-year longitudinal research and indicated that middle-aged PA can reduce mortality and heart attacks in elder life [6]. Despite with the apparent importance of PA, middle-agers may not exercise as required. According to previous survey, 70% of middle-aged people did not meet exercise criteria defined by US federal government [7]. In addition, time spent sitting in middle-aged adults is suggested as too long [8]. As the characteristics of middle-aged life include established own family, clear career direction, and responsibility on children and aging parents [3], people in middle age are busy taking care of families and works, potentially decreasing their PA. Studies have also identified cost and time as barriers for middle-agers to adopt PA [3,9]. These findings suggest that it is important to intervene middle-aged sedentary lifestyle for elder health.

Intervention researchers are utilizing opportunities enabled by technology to design new health interventions. Tailoring an intervention and disseminating it using websites, by email or short message service (SMS) text messages, is considered as a promising health promotion strategy [10]. Recently, middle-agers are increasingly adopting mobile phones. In Nielsen’s report [11] on the global mobile consumer released in 2013, the mobile phone penetration rates in middle-agers reached 40% or higher in some developed countries (eg, United States, United Kingdom, Italy, and South Korea). With the omnipresence and continuity of access, mobile phones therefore become an increasingly essential instrument for revolutionizing intervention strategies [12]. According to a meta-analysis [12], mobile phone apps in the category of PA promotion can measure sports statistics [13] and the number of steps [14], assist self-management (eg, functions with activity diary and reminders) [10]. However, variety of features unnecessarily guarantees the acceptance of users with information technology (IT)–enabled health care applications [15]. The unified theory of acceptance and use of technology (UTAUT) argues that users’ expectations can influence their intention to accept mobile phone apps promoting PA in the context of a voluntary behavior [16]. The expectancy disconfirmation theory also suggests that, when prior-use expectations are matched or exceeded, users feel satisfied and their continuance intention toward product use will be increased [17]. Health care studies also suggest that meeting expectations is crucial not only for enhancing patient satisfaction [18], but also for relieving symptoms and reducing further use of health care resources [19]. Therefore, intervention designers and app developers would benefit in realizing what targeted users expect from using IT-based health care apps in order to deliver necessary functions [20].

To the best of our knowledge, Rabin and Bock [21] may be the first to examine user preferences regarding mobile phone apps related to PA. On the basis of 15 participants, their findings suggest that the most endorsed feature be automatic (and accurate) tracking of steps taken and calories burned, followed by visual tracking on exercise progress and several concrete functionalities (eg, body mass index [BMI] calculators) [21]. Due to the demographical characteristics of their study participants, however, the findings may not be applicable to other populations (eg, middle-agers). Furthermore, as the features were produced based on participant feedbacks, it is desirable to examine features based on a complete theoretical framework, which may contribute to comprehensive understandings both in the feature level and in the theory level.

**Table 1:** Empirical studies with the Kano method.

Authors (year)	Research domain	Product or service type	Research purpose	Product life stage
Chen and Chuang (2008) [26]	Technology	Mobile phone’s body shape and button style	Product performance evaluation	Implementation or testing
Wang and Wu (2014) [27]	Technology	Mobile phone’s core attributes (eg, CPU^a^) and optional attributes (eg, electronic wallet)	Feature classification	Prototype development
Palumbo, Dominici, and Basile (2013) [28]	Technology	Apps function (museum information [eg, opening or closing time], artworks [eg, photo]) and usability (friendly user interface)	Feature classification	Prototype development
Sulisworo and Maniquiz (2012) [29]	Health care	Registration, medical treatment, and physical facilities	Feature classification	Prototype development
Chang and Chang (2013) [30]	Health care	Physical facilities, staff characteristics, medical treatment, and administration	Service performance evaluation	Implementation or testing
Dominici and Palumbo (2013) [31]	Education	E-learning platform	Feature classification	Prototype development
Shahin and Zairi (2009) [32]	Airline	In-flight service, administration, and flight physical facilities	Feature classification	Prototype development

^a^CPU: central processing unit.

### Aims of This Study

The aims of this study were to explore middle-aged adults’ needs on functional features of mobile phone apps promoting PA. First, we created a representative set of 52 design features based on the Coventry, Aberdeen, and London—Refined (CALO-RE) taxonomy of 40 behavior change techniques (BCTs), as this taxonomy can improve the specification of PA interventions and strengthen the scientific study of intervention development [22] and has been applied to examine PA apps [23]. Second, to delve into middle-agers’ needs, this study adopts the Kano method that interprets quality perception as a set of quality categories (eg, attractive, one-dimensional, indifferent, must-be, and reverse) that may influence customer satisfaction [24,25]. Table 1 presents previous studies adopting the Kano method to investigate user satisfaction toward product performance and evaluate the user perceptions of prototypes in design in various application domains (eg, mobile phone design, apps design, and health promotion), warranting applying the Kano method. With our study incorporated with the CALO-RE taxonomy and the Kano method, we can offer insights to realize users’ perception on receiving comprehensive PA interventions via mobile phones and to predict their attitude toward using exercise-promoting apps.

To evaluate the intervention functions offered in mobile phone apps, previous research has applied BCTs to review 64 apps in iTunes and Google Play, and suggested that the apps included 5 BCTs on average [33]. A similar study also downloaded 100 top customer-rated PA apps in the “health and ﬁtness” category of the Apple iTunes and Google Play and suggested that an average of 6.6 BCTs was used per app and most BCTs in the taxonomy were not represented in any app [34]. In addition, for each BCT, this study defines prevalence of a BCT as the percentage of apps implementing the BCT [34]. Although these studies offer insights into how prevalent BCTs are implemented in PA apps, user needs toward these persuasive features remain unexplored, indicating a research gap. Besides, as computer use self-efficacy and the habit of PA can impact users’ acceptance toward mobile phone–based intervention [35,36], whether and how these factors influence the middle-agers’ needs warrants research efforts. To summarize our research questions, this study aims to answer (1) how middle-agers perceive toward mobile phone–based exercise-promoting BCTs in terms of Kano quality categories; (2) whether current mobile phone apps meet middle-agers’ expectations toward mobile phone–based exercise-promoting BCTs; and (3) whether and how the differences across levels of mobile phone self-efficacy (MPSE) and PA influence the quality perception.

## Methods

### Recruitment

This study was designed as a cross-sectional survey conducted from May to August 2015. We recruited participants from two sources. Most of the participants were recruited from those who volunteered to receive physical examinations performed by public health centers in Northern Taiwan. Posters describing study objectivities were displayed in venues where participants attended physical examinations. If an attendant approached recruitment posters, a research assistant introduced him or her to the survey and explained the aim of the study. To increase the number of participants, we also recruited participants in senior communities in Taiwan. All of the participants were told that participation was voluntary, and that all information disclosed would be confidential. All participants provided written consent before being involved in subsequent investigation in which they completed a self-administered questionnaire. Each participant was thanked with a coupon worth of US $6.6.

### Questionnaire Design

The questionnaire includes 4 sections regarding background, MPSE (10 items), PA (29 items), and quality perception toward BCTs via mobile phone (104 items). MPSE refers to mobile phone users’ confidence to undertake specific tasks (eg, interacting with PA apps) [37]. This construct should be included in the research model, as eHealth studies are recommended to avoid generalizability issues arisen from assuming the reference population to be skilled in using a computer [35]. The 10-item Computer Self-Efficacy Scale was adapted for use in this study [38]. The items were modified to fit our research context of using mobile phone apps (eg, “if there was no one around to tell me what to do as I go, I could use mobile phone apps to manage PA” and “if I had never used a mobile phone app to manage PA before, I am confident of using such an app”). All items were scored on a 5-point Likert scale from “strongly disagree” to “strongly agree,” with higher scores representing higher MPSE. We used tertiary split to divide this variable into three levels to examine the quality perception toward design features in each level of MPSE. The 10-item scale had high internal consistency (Cronbach alpha=.922).

Physical activity level may influence the effect of PA intervention [36], suggesting the necessity of incorporating this important variable. To measure PA level, the International Physical Activity Questionnaire (IPAQ) was developed to assess the frequency and duration of vigorous intensity, moderate intensity, and walking activity. This questionnaire has two versions available: the International Physical Activity Questionnaire-long form (IPAQ-LF) and the International Physical Activity Questionnaire-short form (IPAQ-SF). Several studies have suggested that the IPAQ has acceptable reliability and validity [39-42], including a 12-country study [43]. Since its invention, the IPAQ has become one of the most widely used PA questionnaires [41]. This scale, as a proxy for PA level, was used to examine the effect of technology-mediated intervention (eg, websites and email [44,45]), and to predict physical function (eg, pain facilitatory and inhibitory function [46]) and mental health (eg, risks of persistent late-life depression [47]).

This study adopted the long form version of IPAQ-Taiwan, modifying the original IPAQ-LF version with cultural adaptation. The IPAQ-Taiwan developers reported that the long form version had a content validity indice of .992, suggesting high language equivalence with the original English version. The consistency value for the English and Chinese versions in terms of intraclass correlation coefficients were .945, also indicating the appropriateness of reliability [42]. According to the usefulness guidelines suggested by Heesch et al [48], the 28-item IPAQ-Taiwan defines vigorous PA and moderate PA in “Introduction” section, followed by 5 sections requesting participants of their time spent in PA during the past 7 days in terms of different activity classes (eg, work-related and transport-related activity). The IPAQ-Taiwan clarifies activities mentioned as examples in the questionnaire, consistent with another suggestion by Heesch et al [48]. As with the original IPAQ, PA time in three levels (ie, vigorous-level, moderate-level, and walking) of four domains (ie, work, transport, domestic and garden, and leisure) was filled by participants. We followed the IPAQ group’s scoring protocol to assess participants’ level of PA, which can be found in Multimedia Appendix 1.

The questionnaire measuring quality perception toward BCTs via mobile phone was developed in this study. On the basis of the CALO-RE taxonomy, two of the authors developed 52 design features of mobile phone apps targeted at promoting PA, as illustrated in Table 2. If mobile phones can implement a BCT with more than one way, each way was designated as a design feature. For example, three design features (eg, contextual cues [A31], location cues [A32], and people cues [A33]) were derived from the BCT of using contextual cues.

For each design feature, a functional question asks participants’ feelings in the case of fulfillment of the feature, and a dysfunctional question asks participants’ feelings in the case of nonfulfillment of the feature [26,30,49]. Participants answered each question by choosing one of the five options: “Satisfied,” “It should be that way,” ” I am indifferent,” “I can live with it,” and “Dissatisfied.” For a specific participant, his or her quality perception toward the design feature can be determined by looking up the Kano evaluation matrix (Table 3) with the functional answer and the dysfunctional answer. There are five possible quality categories: *attractive* (A), *one-dimensional* (O), *must-be* (M), *reverse* (R), and *indifferent* (I) [49]. Assuming a nonlinear relationship between product performance and customer satisfaction [25,50,51], the Kano method defines an attribute is *attractive* (A) if an increase in the performance of an attribute enhances customer satisfaction, whereas a decrease in performance does not lead to dissatisfaction; an attribute is *one-dimensional* (O) if an increase in the performance of an attribute enhances customer satisfaction, whereas a decrease in performance also increases dissatisfaction; an attribute is *must-be* (M) if an increase in the performance does not increase satisfaction, but a deteriorating performance increases dissatisfaction; and an attribute is *indifferent* (I) if neither an increasing performance nor a decreasing performance can affect satisfaction. The definitions of reverse and questionable attributes can be found in [25,50]. The quality perception toward a design feature over a sample can be determined by selecting the highest frequency of quality categories for all the participants in the sample [49].

**Table 2:** A representative set of 52 design features based on the Coventry, Aberdeen, and London—Refined (CALO-RE) taxonomy.

Design features of exercise-promotion apps	Code
Apps provide information on consequences of exercise in general.	A1
Apps provide information on customized consequences of exercise.	A2
Apps provide information about others’ approval of my exercise.	A3
Apps provide information about others’ exercise status.	A4
Apps provide information about avoided movement in exercise.	A5
Apps help set exercise goals.	A6
Apps help set graded tasks by decomposing goals.	A7
Apps prompt review of exercise goals.	A8
Apps can check the extent to which previously set exercise goals were achieved.	A9
Apps remind me to record my exercise behavior.	A10
Apps can record my exercise behavior automatically.	A11
Apps can set the health goals to be achieved by exercise.	A12
Apps can prompt review of health goals.	A13
Apps can check the extent to which my expected goals were achieved.	A14
Apps remind me to keep records of my exercise outcome.	A15
Apps can automatically record my exercise outcome.	A16
Apps can assist me in detailed exercise planning.	A17
Apps can remind me to think about potential barriers in exercise planning.	A18
Apps can remind me to identify the ways of overcoming potential barriers when exercise planning.	A19
Apps prompt rewards contingent on effort toward exercise preparation.	A20
Apps provide rewards contingent on successful exercise.	A21
Apps provide graded use of contingent rewards over time.	A22
Apps prompt generalization of exercise.	A23
Apps remind me of past successful experience of exercise.	A24
Apps provide exercise records.	A25
Apps check the discrepancy between exercise performance and the set goals.	A26
Apps provide me with data about the discrepancy between my exercise performance and others’.	A27
Apps provide information on where and when to do the exercise.	A28
Apps provide instructions on how to do the exercise by text or voice.	A29
Apps show how to do the exercise through visual demonstrations.	A30
Apps can set context cues which remind me to exercise.	A31
Apps can set location cues which remind me to exercise.	A32
Apps can set people cues which remind me to exercise.	A33
Apps remind me to alter environment in ways so that it is more supportive of the exercise.	A34
Apps create the exercise goals as agreed behavioral contract.	A35
Apps prompt me to rehearse or repeat the exercise behavior numerous times.	A36
Exercise reminders are gradually reduced in intensity, duration, and frequency over time.	A37
Apps facilitate social comparison.	A38
Apps make it easy to elicit social support to my exercise from other people.	A39
Apps remind me to focus on partners who are the exercise role models.	A40
Apps facilitate the discussion with exercise role models.	A41
Apps induce perceptions of future regret about not doing exercise.	A42
Apps provide risk information which evokes a fearful response.	A43
Apps prompt self-talk to encourage, support, and maintain exercise.	A44
Apps prompt mental imagery (to imagine initiating or maintaining exercise is easy).	A45
Apps provide strategies in advance to avoid sustainability problem of exercise.	A46
Apps provide stress management to reduce anxiety to facilitate the performance of the exercise.	A47
Apps remind me to attend motivational interviewing which can minimize resistance and resolve ambivalence to change.	A48
Apps assist time management to make time for exercise.	A49
Apps provide general communication skills training.	A50
Apps stimulate anticipation of future rewards.	A51
Apps can set exercise time reminders.	A52

**Table 3:** The Kano evaluation matrix.

Quality attribute	Dysfunctional answer					
Functional answer		Satisfied	It should be that way	I am indifferent	I can live with it	Dissatisfied
	Satisfied	Q^a^	A^b^	A	A	O^c^
	It should be that way	R^d^	I^e^	I	I	M^f^
	I am indifferent	R	I	I	I	M
	I can live with it	R	I	I	I	M
	Dissatisfied	R	R	R	R	Q

^a^Q: questionable.

^b^A: attractive.

^c^O: one-dimensional.

^d^R: reverse.

^e^I: indifferent.

^f^M: must-be.

### Statistical Analysis

The analysis began with excluding invalid responses. As the chronological definition of middle age varies in existing studies (eg, 40-59 years [2], 45-74 years [52], and 40-60 years [53]), we included responses only from participants aged between 40 and 69 years. Next, missing values were identified, which was followed with a value substitution procedure. As the variables with more than one missing value were all in the Kano questionnaire, the importance of the Kano variables suggested the use of multiple imputations. We opted to include all the nonmissing variables as predictors in the initial imputation model. With multinomial logistic regression, the variables with significant predictability were identified. As suggested in [54], we used three imputations for every missing value. As the Kano method determines the categories with the largest number of votes, we created the final set of winning categories by forming the union of winning categories in three imputations. The analysis revealed that the winning categories in each imputation were the same, which may be attributed to the small proportions of missing values. After the multiple imputation procedure, all the data entered the Kano analysis.

#### The Kano Analysis

The Kano method determines the highest frequency of quality categories in a design feature as the winning category, as these categories represent the dominant customer view [55]. Previous study applying the Kano method suggested the use of two additional measures, category strength, and total strength, to determine whether there exists more than one attribute that dominates [56]. Category strength is deﬁned as “the extent of how ﬁrmly the participants felt that an item was in one category or another” [56]. We calculated the difference (in percentage) between the highest and the second highest categories to measure category strength. A category strength value greater than 6% indicates a statistical difference between the highest and the second highest categories [56]. Total strength is defined as the total proportion of positive attributes (ie, *attractive* [A], *one-dimensional* [O], and *must-be* [M]). According to [56], if the category strength of an attribute were lower than 6% and the total strength exceeded 60%, then it could be statistically impossible to classify the attribute in one category or another (referred to as the Lee and Newcomb rule hereafter). Because 60% could be determined arbitrarily, such an attribute would fall into the mixed (X) category [55], indicating that a design feature may turn out to be determined as multiple categories. We adopted an aggressive position in which design features deserve recommendations as long as the categorization results include any positive category. This position emphasizes the importance of customer demands while also creating opportunities of adding value to mobile phone–based exercise promotion.

#### Two-Factor Analysis

To realize the predictability of two independent variables, we created a multinomial regression model for each of those design features. By regressing quality perception on PA level and MPSE, the fit of the models was examined and the likelihood ratio tests were conducted to ascertain the significance of predictors.

### Institutional Review Board (IRB)

Ethical approval was granted by the Institutional Review Board (IRB) and Chang Gung Memorial Hospital (CGMH) (103-2125B and 104-3029C). Permission for data collection was also obtained from the officials of the public health centers. The participants were informed about the study, its importance, and confidentiality of the information collected. They were also told that they owned rights to leave the study at any time before signing their written consent for participation in the study. All participants’ data are maintained in a secure manner by separating participants’ identifiers and associated data, as recommended by the IRB.

## Results

### Sample Demographics

The sample size was 105. The participants had an average age of 55.7 years. The sex ratio of participants in the nonmissing responses was 53:50. Most participants (73/105; 69.5%) had at least a senior high school education and more than a half in the nonmissing responses (53/98; 54%) had an annual income of NT $720,000 (US $23,630) or more. More than half of the participants (n=58) used mobile phone apps longer than half an hour per day, and about one-fifth (n=20) played apps longer than 2 h. Demographic data are presented in Table 4. We also asked the participants about their most frequently used mobile phone apps. The result showed that the LINE (n=62) and Facebook apps (n=11) were respectively ranked in the first and second places, suggesting the popularity of social networking apps.

### Kano Analysis

This section proposes the analysis results obtained in conducting Kano analysis on the total sample and the subsamples by PA and by MPSE. When leading categories had close votes, we used the Lee and Newcomb rule to determine whether to list multiple winners. When winning categories of a design feature included the *indifferent* category and at least one positive category (ie, *attractive*, *one-dimensional*, *must-be*), only positive categories were described in-text, as positive categories are more informative to app developers. Nevertheless, we reported in Tables 5-10 with an expression X(P, I) to indicate a tie between a positive category P and the *indifferent* category (I). To make this paper concise, design features are not shown in Tables 5-10 unless their categorization results include at least one positive category, whereas complete results can be found in Multimedia Appendix 2.

Table 5 reports the categorization results based on the responses from all participants who filled out the Kano questionnaire (n=103). It was found that 51 of the 52 design features were classified as *indifferent*, suggesting that these 51 design features did not interest the subjects in the total sample. One design feature (A30: visual demonstrations) may be categorized as *attractive (28)*, despite with a tie with the *indifferent* category.

The categorization results in the subsamples of PA levels were based on 102 valid responses from those participants who completed both the Kano questionnaire and the IPAQ. We first examined the high-PA participants and found that all of the 52 design features were categorized as *indifferent*. As no design features with a smaller-than 0.06 category strength had a total strength larger than 60%, we had sufficient confidence that these designs did not motivate this subsample.

Table 6 shows the categorization results of the medium-PA participants. For this specific subsample, mobile phone apps offered limited incentives to use. It was found that 46 of the 52 design features were determined as one category of *indifferent*. A10 (reminding to record PA) was categorized as *must-be (7)*, suggesting that the participants with medium-PA considered as granted the design feature of reminding to record exercise behavior. Five other design features had close proportions in the *indifferent* category and a positive category, including A5 (offering movements to be avoided) categorized into *one-dimensional (5)*; A11 (automatically record physical activity [PA]) into *attractive (6)*; A20 (contingent reward for exercise preparation) into *must-be (5)*; A23 (prompt generalization of exercise) into *one-dimensional (6)*; and A29 (exercise instruction with text or voice) into *one-dimensional (6)*. Most of the functions needed are related to information provision and behavioral facilitation.

**Table 4:** Descriptive statistics on sample demographics (N=105).

Variable		n (%)
**Gender**		
	Male	53 (50.5)
	Female	50 (47.6)
	Missing	2 (1.9)
**Education**		
	Junior high school or less	30 (28.6)
	Senior high school	33 (31.4)
	Bachelor’s degree	39 (37.1)
	Graduate degree	1 (1.0)
	Missing	2 (1.9)
**Employment**		
	Employed	74 (70.5)
	Unemployed or retired	29 (27.6)
	Missing	2 (1.9)
**Marital status^a^**		
	Married	84 (80.0)
	Widowed	7 (6.7)
	Divorced	8 (7.6)
	Not married	3 (2.9)
	Missing	3 (2.9)
**Monthly household income^a^**		
	≤NT $39,999 (US $1313)	22 (21.0)
	≤NT $49,999 (US $1641)	12 (11.4)
	≤NT $59,999 (US $1969)	11 (10.5)
	≤NT $69,999 (US $2297)	9 (8.6)
	≤$NT $79,999 (US $2626)	8 (7.6)
	≤NT $89,999 (US $2954)	12 (11.4)
	>NT $90,000 (US $2955)	24 (22.9)
	Missing	7 (6.7)
**Daily app using time**		
	≤30 min	43 (41.0)
	≤120 min	38 (36.2)
	≤240 min	16 (15.2)
	>240 min	4 (3.8)
	Missing	4 (3.8)

^a^Percentages may not add up to 100 due to rounding.

**Table 5:** Categorizing design features by the total sample (n=103).

Design features	Frequency of design feature						Category strength (%)	Total strength (%)	Classification results
	A	M	O	I	R	Q			
A30	28	15	23	33	1	3	5	64	X(I, A)^a^

^a^X(C_1_, C_2_) indicates that a design feature had close proportions in two categories of C_1_ and C_2_.

**Table 6:** Categorizing design features by medium physical activity participants (n=18).

Design features	Frequency of design feature						Category strength (%)	Total strength (%)	Classification results
	A	M	O	I	R	Q			
A5	4	3	5	6	0	0	6	67	X(I, O)^a^
A10	4	7	2	5	0	0	11	72	M
A11	6	5	1	6	0	0	0	67	X(A, I)^a^
A20	4	5	3	6	0	0	6	67	X(I, M)^a^
A23	2	3	6	7	0	0	6	61	X(I, O)^a^
A29	4	1	6	7	0	0	6	61	X(I, O)^a^

^a^X(C_1_, C_2_) indicates that a design feature had close proportions in two categories of C_1_ and C_2_.

In contrast, low-PA users need much more support from mobile phone–based apps. Table 7 shows the categorization results of low-PA participants. In total, 31 design features were categorized only as *indifferent*. It was found that 6 design features (A1: general consequences of exercise; A2: customized consequences of exercise; A13: browse health goals; A22: contingent rewards with grading; A25: PA history; and A26: comparing actual PA with PA goal) were categorized as *must-be*, and 1 design feature (A14: compare actual health outcomes with health goals) was categorized into *one-dimensional*. The remaining 14 design features were categorized into more than one category according to the Lee and Newcomb rule. Among these 14 design features, 7 were classified into only positive categories, including A7 (help goal decomposition: *one-dimensional* (7) and *must-be* (6)), A10 (remind to record PA: *one-dimensional* (7) and *must-be* (6)), A15 (remind to record health outcomes: *one-dimensional* (7) and *must-be* (7)), A21 (contingent reward for exercise practice: *one-dimensional* (7) and *must-be* (7)), A23 (prompt generalization of exercise: *one-dimensional* (7) and *must-be* (6)), A29 (exercise instruction with text or voice: *attractive* (7) and *must-be* (7)), and A30 (visual demonstration: *attractive* (7) and *must-be* (7)). The other 7 design features were determined as multiple categories including *indifferent*: A6 (set PA goals: *must-be* (7)), A8 (browse PA goals: *must-be* (7)), A9 (check goal conversions: *must-be* (7)), A12 (set health goals: attractive (5), *must-be* (6), *one-dimensional* (5)), A20 (contingent reward for exercise preparation: *one-dimensional* (6)), A24 (remind past success: *attractive* (6) and *must-be* (6)), and A52 (reminding to PA: *attractive* (6), *must-be* (7)). Low-PA users required assistance in goal management and time management. Furthermore, 6 motivational techniques (ie, A1, A2, A20, A21, A22, and A24) were considered either as *must-be* or as *one-dimensional*, suggesting the importance of extrinsic motivation to low-PA users.

A further analysis on the 21 design features revealed that only two (ie, A14 and A20) were not categorized as *must-be*, and all the 5 design features categorized into *attractive* were also categorized as *must-be*. Therefore, this finding suggested that these designs may be more of necessity than of attractiveness to low-PA participants.

The categorization results across levels of MPSE were based on 102 valid responses from those participants who completed both the Kano questionnaire and the MPSE questionnaire. Table 8 presents the categorization results of participants with high self-efficacy. A total of 4 design features (A23: prompt generalization of exercise; A26: comparing actual PA with PA goal; A29: exercise instruction with text or voice; and A30: visual demonstrations) were categorized as *one-dimensional*. It was found that 10 design features were categorized into more than one category including *indifferent*, including A2 (customized consequences of exercise: *must-be* (10)), A10 (remind to record PA: *must-be* (10)), A12 (set health goals: *one-dimensional* (11)), A13 (browse health goals: *one-dimensional* (10)), A14 (compare actual health outcomes with health goals: *one-dimensional* (11)), A15 (remind to record health outcomes: *one-dimensional* (10)), A21 (contingent reward for exercise practice: *one-dimensional* (10)), A22: (contingent rewards with grading: *one-dimensional* (14)); A25 (PA history: *one-dimensional* (12)), and A52 (reminding to PA: *one-dimensional* (10)). A breakdown analysis on the 14 design features revealed that, except with 2 design features (ie, A2 and A10) categorized as *must-be*, the other 12 design features were all *one-dimensional*. Therefore, this analysis suggested that users with high MPSE would consider these designs as more satisfactory as the mobile phone apps perform better in terms of these design features. The remaining 38 design features were all categorized as *indifferent*.

**Table 7:** Categorizing design features by low physical activity participants (n=22).

Design features	Frequency of design feature						Category strength (%)		Total strength (%)		Classification results	
	A	M	O	I	R	Q						
A1	1	11	2	8	0	0		14		64		M
A2	1	8	4	8	0	1		0		59		M
A6	2	7	5	8	0	0		5		64		X(I, M)^a^
A7	3	6	7	5	1	0		5		73		X(O, M)^a^
A8	4	7	4	7	0	0		0		68		X(M, I)^a^
A9	3	7	4	8	0	0		5		64		X(I, M)^a^
A10	3	6	7	5	0	1		5		73		X(O, M)^a^
A12	5	6	5	5	1	0		5		73		X(M, A, O, I)^a^
A13	3	8	5	6	0	0		9		73		M
A14	3	6	8	3	1	1		9		77		O
A15	4	7	7	3	0	1		0		82		X(M, O)^a^
A20	4	5	6	7	0	0		5		68		X(I, O)^a^
A21	4	7	7	3	0	1		0		82		X(M, O)^a^
A22	5	8	6	3	0	0		9		86		M
A23	4	6	7	4	1	0		5		77		X(O, M)^a^
A24	6	6	4	6	0	0		0		73		X(A, M, I)^a^
A25	4	8	5	5	0	0		14		77		M
A26	4	9	5	4	0	0		18		82		M
A29	7	7	5	3	0	0		0		86		X(A, M)^a^
A30	7	7	5	3	0	0		0		86		X(A, M)^a^
A52	6	7	3	6	0	0		5		73		X(M, A, I)^a^

^a^X(C_1_, C_2_, ..., C_n_) indicates that a design feature had close proportions in the categories of C_i_, 1≤ *i* ≤ *n*.

Table 9 presents the categorization results of medium MPSE participants. A total of 46 design features were categorized as *indifferent* and 4 design features (ie, A15: remind to record health outcomes; A22: contingent rewards with grading; A25: PA history; and A52: reminding to PA) were categorized as *must-be*. A23 (prompt generalization of exercise) and A26 (comparing actual PA with PA goal) were determined as a tie with two categories of *indifferent* and *must-be* (ie, A23: *must-be* (10); A26: *must-be* (13)). This analysis suggested that this specific subsample had weak intention to use mobile phone apps to promote exercise and tended to consider the functions as of necessity.

**Table 8:** Categorizing design features by high self-efficacy participants (n=35).

Design features	Frequency of design feature							Category strength (%)		Total strength (%)		Classification results
	A	M	O	I	R	Q						
A2	6	10	7	11	0	1	3		66		X(I, M)^a^	
A10	6	10	8	10	0	1	0		69		X(M, I)^a^	
A12	6	5	11	12	0	1	3		63		X(I, O)^a^	
A13	7	7	10	9	0	2	3		69		X(O, I)^a^	
A14	6	6	11	10	0	2	3		66		X(O, I)^a^	
A15	5	8	10	10	0	2	0		66		X(O, I)^a^	
A21	7	5	10	11	0	2	3		63		X(I, O)^a^	
A22	4	3	14	12	1	1	6		60		X(O, I)^a^	
A23	6	3	14	10	0	2	11		66		O	
A25	6	4	12	11	0	2	3		63		X(O, I)^a^	
A26	5	6	13	9	0	2	11		69		O	
A29	7	3	15	8	0	2	20		71		O	
A30	9	3	13	7	1	2	11		71		O	
A52	6	6	10	12	0	1	6		63		X(I, O)^a^	

^a^X(C_1_, C_2_) indicates that a design feature had close proportions in two categories of C_1_ and C_2_.

**Table 9:** Categorizing design features by medium self-efficacy participants (n=32).

Design features	Frequency of design feature							Category strength (%)		Total strength (%)		Classification results
	A	M	O	I	R	Q						
A15	3	12	4	12	0	1	0		59		M	
A22	7	12	5	8	0	0	13		75		M	
A23	6	10	4	10	2	0	0		63		X(M,I)^a^	
A25	6	13	3	10	0	0	9		69		M	
A26	4	13	3	12	0	0	3		63		X(M,I)^a^	
A52	7	12	3	10	0	0	6		69		M	

^a^X(C_1_, C_2_) indicates that a design feature had close proportions in two categories of C_1_ and C_2_.

**Table 10:** Categorizing design features by low self-efficacy participants (n=35).

Design features	Frequency of design feature							Category strength (%)		Total strength (%)		Classification results
	A	M	O	I	R	Q						
A30	12	4	7	12	0	0	0		66		X(A, I)^a^	

^a^X(C_1_, C_2_) indicates that a design feature had close proportions in two categories of C_1_ and C_2_.

Table 10 presents the categorization results by the low MPSE subsample. Only A30 (visual demonstration) was categorized as both *attractive* and *indifferent*. All of the remaining designs were *indifferent*. This subsample exhibited low interest in those design features to increase PA.

Next, we compared users’ demands with mobile phone–based BCTs and the supply in the mobile phone apps market. Table 11 compares the categorization results across the total sample and subsamples. If the quality categories in a cell are shown with a superscript *i*, this means that these categories are tied with the *indifferent* category. According to [34], the rightmost field in Table 11 provides the prevalence of particular BCTs delivered by popular mobile phone apps. The range of prevalence numbers of demanded design features was [0%, 49%] with an average of 20% (4.3/21). The two top-ranked features (ie, social support [A39, 79%] and social approval [A3, 64%], also listed in Table 11) were available in more than half of the apps inspected. However, both features were considered as *indifferent* by our sample and in all of the subsamples, either by PA level or by MPSE. Exercise instruction (A29, 49%) and visual demonstration (A30, 47%), ranked in the next two places, were considered as needed. On the other hand, design features that can contribute to fulfill users’ needs across different user groups are available in less than 40% of the apps inspected. For example, prompting exercise generalization (A23, 0%) is valuable to four subsamples of middle-agers, but was not found in the inspected apps. Prompts or cues (A52) can create value to three subsamples (ie, low-PA, high-MPSE, and medium-MPSE), but only 35% of the apps offered this feature. Table 11 also showed that reminding to record PA (A10, 29%), reminding to record health outcomes (A15, 22%), and contingent rewards with grading or shaping (A22, 1%) were unavailable in most of the apps but each feature was needed in three subsamples. Furthermore, 5 design features were needed by two subsamples but the prevalence numbers were not larger than 30% (eg, browse health goals [A13, 6%] and contingent reward for exercise practice [A21, 3%]). These findings suggest that current supply of exercise-promoting features in mobile phone apps might not fully match middle-aged adults’ needs. The gaps represent strategic opportunities for app designers to fulfill the needs of customers with customized characteristics.

### Two-Factor Analysis

To realize the effects of PA level and MPSE on quality categorization (ie, the third research question), we created one multinomial logistic regression model for each design feature. To avoid zero frequency, we combined the *attractive* and *one-dimensional* categories into a new *valued* category. The *must-be* category remained intact, whereas all of the other instances were entered into the *indifferent* category. The left-hand part of Multimedia Appendix 3 provides the Pearson and deviance statistics for model fitting. As the deviance statistics of three models (ie, A20, A32, and A43) were significant, which suggested significant deviation from observations to predicted values, we excluded these models from the likelihood ratio tests that followed.

The remaining 49 models were examined with the likelihood ratio tests to ascertain the significance of predictors to the models. The chi-square statistics and the significance of coefficients for two predictor variables (ie, MPSE and PA level) were listed in the right-hand part of the Multimedia Appendix 2. MPSE was significant in 14 models (ie, A3, A6, A8, A10, A13, A18, A22, A25, A26, A29, A35, A41, A45, and A48), whereas only 1 (ie, A3) was found as significant for PA level. This analysis revealed that combining MPSE and PA could significantly predict quality categorization on A3, whereas only MPSE was a significant predictor predicting other 13 design features.

To delve into the effect of different levels of predictors, we analyzed the effects of coefficients on design satisfaction with regard to each of the 14 design features. We first analyzed the effect of PA on categorizing A3 (social approval). With the medium-PA group as the baseline, neither the estimates for high-PA (*b*=−0.550, Wald χ^2^_1_=0.5 odds ratio, OR 0.58 [95% CI 0.12-2.82], *P*=.50) nor the estimates for low-PA (*b*=−1.453, Wald χ^2^_1_=1.4; OR 0.23 [95% CI 0.02-2.53], *P*=.24) for the *must-be* category (compared with the *indifferent* category) was significant. For the *valued* category (compared with the *indifferent* category), the estimates for high-PA (*b*=19.703, Wald χ^2^_1_=540.5; OR 3.60E-8 [95% CI 6.85E-7 to 1.90E-9], *P*<.001) was significant. This suggested that high-PA participants were more likely to categorize A3 into *attractive* or *one-dimensional* than the medium-PA group.

We next turned to assess the estimates of MPSE coefficients. Table 12 presents the *b* values, the Wald statistics, the values of significance of testing the estimates across the MPSE levels, and 95% CI for OR for the *must-be* category compared with the *indifferent* category. The analysis revealed that, compared with their low MPSE counterparts, medium MPSE participants more likely categorized 5 design features as *must-be* than as *indifferent*. More specifically, this medium MPSE group considered the following features as *must-be*: A6 (set PA goals: *b*=−1.712, Wald χ^2^_1_=5.4; OR 0.18 [95% CI 0.04-0.77], *P*=.02), A22 (contingent rewards with grading: *b*=−1.790, Wald χ^2^_1_=5.7; OR 0.17 [95% CI 0.04-0.72], *P*=.02), A25 (PA history: *b*=−1.590, Wald χ^2^_1_=5.5; OR 0.20 [95% CI 0.05-0.77], *P*=.02), A26 (comparing actual PA with PA goal: *b*=−1.370, Wald χ^2^_1_=4.2; OR 0.25 [95% CI 0.07-0.94], *P*=.04), and A45 (prompt mental imagery: *b*=−2.770, Wald χ^2^_1_=6.1; OR 0.06 [95% CI 0.01-0.56], *P*=.01). Besides, compared with high-MPSE participants, medium MPSE participants more likely considered A22 (contingent rewards with grading) as *must-be* than as *indifferent* (*b*=−1.932, Wald χ^2^_1_=5.5; OR 0.15 [95% CI 0.03-0.73], *P*=.02), as illustrated in Table 13. The OR indicates that the change in the odds of categorizing A22 as *must-be* compared with *indifferent* is .15 as the MPSE level changes from medium to high, which suggests that this design feature was more likely categorized to *must-be* (compared with *indifferent*) by medium MPSE participants than by high-MPSE participants. As Table 14 indicates that no design feature was found with a significant regression coefficient, this analysis found no evidence to argue that the categorization likelihood differs between the high-MPSE and low-MPSE subsamples.

**Table 11:** Positive quality categories by design features and participant characteristics.

Design feature	Total sample	PA^a^ (H)	PA (M)	PA (L)	MPSE^b^ (H)	MPSE (M)	MPSE (L)	Prevalence (%) [34]
A1 (general consequences of exercise)^c^				M				2
A2 (customized consequences of exercise)^c^				M	M^g^			2
A3 (social approval)								64
A5 (offering movements to be avoided)^f^			O^g^					N/A^h^
A6 (set PA goals)				M^g^				36
A7 (help goal decomposition)				(O, M)				33
A8 (browse PA goals)				M^g^				17
A9 (check goal conversions)				M^g^				8
A10 (remind to record PA)			M	(O, M)	M^g^			29
A11 (automatically record PA)			A^g^					29
A12 (set health goals)				(A, O, M)^g^	O^g^			17
A13 (browse health goals)^d^				M	O^g^			6
A14 (compare actual health outcomes with health goals)^d^				O	O^g^			6
A15 (remind to record health outcomes)				(O, M)	O^g^	M		22
A20 (contingent reward for exercise preparation)^f^			M^g^	O^g^				N/A
A21 (contingent reward for exercise practice)				(O, M)	O^g^			3
A22 (contingent rewards with grading or shaping)^e^				M	O^g^	M		1
A23 (prompting exercise generalization)^f^			O^g^	(O, M)	O	M^g^		0
A24 (remind past success)				(A, M)^g^				4
A25 (PA history)				M	O^g^	M		42
A26 (comparing actual PA with PA goal)				M	O	M^g^		42
A29 (PA instruction with text or voice)			O^g^	(A, M)	O			49
A30 (visual demonstration)	A^g^			(A, M)	O		A^g^	47
A39 (social support)								79
A52 (reminding to PA)				(A, M)^g^	O^g^	M		35
Number of features	1	0	6	21	14	6	1	

^a^PA: physical activity.

^b^MPSE: mobile phone self-efficacy.

^c^Corresponding to information about health consequences in [34].

^d^Corresponding to review outcome goals in [34].

^e^Corresponding to reward approximation in [34].

^f^No observation reported in [34].

^g^Tied with the *indifferent* category.

^h^N/A: not applicable.

**Table 12:** Parameter estimates of change of mobile phone self-efficacy (MPSE) from medium to low (*must-be* over *indifferent*).

Design feature	Mobile phone self-efficacy (H vs L)					95% CI for odds ratio (lower bound-upper bound)
	*b*	Wald	Significance	Odds ratio		
A3	−0.592	0.4	.54	0.55	0.08-3.70	
A6	−1.712	5.4	.02	0.18	0.04-0.77	
A8	−0.778	1.2	.27	0.46	0.12-1.84	
A10	−0.673	1.1	.30	0.51	0.14-1.81	
A13	−0.995	2.3	.13	0.37	0.10-1.34	
A18	−0.551	0.5	.50	0.58	0.12-2.88	
A22	−1.790	5.7	.02	0.17	0.04-0.72	
A25	−1.590	5.5	.02	0.20	0.05-0.77	
A26	−1.370	4.2	.04	0.25	0.07-0.94	
A29	−0.981	1.8	.18	0.38	0.09-1.58	
A35	−0.655	0.3	.61	0.52	0.04-6.21	
A41	−1.311	1.2	.28	0.27	0.03-2.86	
A45	−2.770	6.1	.01	0.06	0.01-0.56	
A48	−1.533	3.1	.08	0.22	0.04-1.19	

**Table 13:** Parameter estimates of change of mobile phone self-efficacy (MPSE) from medium to high (*must-be* over *indifferent*).

Design feature	Mobile phone self-efficacy (H vs L)				95% CI for odds ratio (lower bound-upper bound)
	*b*	Wald	Significance	Odds ratio	
A3	0.494	0.4	.55	1.64	0.32-8.38
A6	−0.438	0.5	.48	0.65	0.19-2.16
A8	−0.144	0.0	.83	0.87	0.24-3.12
A10	0.210	0.1	.73	1.23	0.38-4.03
A13	−0.119	0.0	.85	0.89	0.25-3.13
A18	−20.685	N/A^a^	N/A	1.04E-9	1.04E-9 to 1.04E-9
A22	−1.932	5.5	.02	0.15	0.03-0.73
A25	−1.389	3.6	.06	0.25	0.06-1.05
A26	−0.558	0.7	.41	0.57	0.15-2.16
A29	−0.841	1.1	.31	0.43	0.09-2.15
A35	0.224	0.0	.83	1.25	0.16-9.71
A41	−19.646	N/A	N/A	2.94E-9	2.94E-9 to 2.94E-9
A45	−1.010	2.1	.15	0.36	0.09-1.45
A48	−21.029	N/A	N/A	7.37E-10	7.37E-10 to 7.37E-10

^a^N/A: not applicable.

**Table 14:** Parameter estimates of change of mobile phone self-efficacy (MPSE) from low to high (*must-be* over *indifferent*).

Design feature	Mobile phone self-efficacy (H vs L)					95% CI for odds ratio (lower bound-upper bound)
	*b*	Wald	Significance	Odds ratio		
A3	1.085	1.4	.24	2.96	0.48-18.11	
A6	1.274	2.7	.10	3.58	0.78-16.73	
A8	0.634	0.7	.41	1.89	0.42-8.37	
A10	0.882	1.7	.19	2.42	0.64-9.06	
A13	0.876	1.5	.22	2.40	0.59-9.78	
A18	−20.134	N/A^a^	N/A	1.80E-9	0.00-0.00	
A22	−0.142	0.0	.87	0.87	0.16-4.85	
A25	0.201	0.1	.80	1.22	0.27-5.64	
A26	0.812	1.2	.27	2.25	0.53-9.56	
A29	0.140	0.0	.88	1.15	0.20-6.61	
A35	0.880	0.5	.49	2.41	0.20-28.68	
A41	−18.335	N/A	N/A	1.09E-8	0.00-0.00	
A45	1.761	2.3	.13	5.82	0.59-57.22	
A48	−19.496	N/A	N/A	3.41E-9	0.00-0.00	

^a^N/A: not applicable.

We next turned to analyze the effect of MPSE on the relative likelihoods of the *valued* category versus the *indifferent* category, as illustrated in Tables 15-17. In comparing the high group to the medium group, MPSE was able to predict the relative likelihoods with regard to 5 design features, as indicated in Table 15. Specifically, the high-group is more likely considered as *valued* in A3 (social approval: *b*=2.622, Wald χ^2^_1_=5.6; OR 13.77 [95% CI 1.57-120.51], *P*=.02), A8 (browse PA goals: *b*=1.540, Wald χ^2^_1_=5.8; OR 4.66 [95% CI 1.33-16.37], *P*=.02), A10 (reminding to record PA: *b*=1.522, Wald χ^2^_1_=5.0; OR 4.58 [95% CI 1.21-17.39], *P*=.03), A13 (browse health goals: *b*=1.673, Wald χ^2^_1_=6.4; OR 5.33 [95% CI 1.47-19.37], *P*=.01), A35 (PA fulfilled as a contract: *b*=20.051, Wald χ^2^_1_=719.9; OR 5.11E-8 [95% CI 1.18E-8 to 2.21E-9], *P*<.001), and A41 (Talk to role models: *b*=1.573, Wald χ^2^_1_=4.9; OR 4.82 [95% CI 1.19-19.49], *P*=.03). The high-group also categorized A26 as *valued*, compared with the low-group, as suggested by the regression coefficient and the Wald statistic (comparing actual PA with PA goal: *b*=1.237, Wald χ^2^_1_=4.8; OR 3.444 [95% CI 1.15-10.35], *P*=.03) as shown in Table 16. Moreover, the low group, compared with their medium counterpart, had a relative advantage in categorizing A10 (remind to record PA) into *valued* rather than *indifferent* (*b*=1.324, Wald χ^2^_1_=4.0; OR 3.76 [95% CI 1.02-13.85], *P*=.047). The OR indicates that the change in the odds of categorizing A10 as *valued* compared with *indifferent* is 3.76 as the MPSE level changes from medium to low, which is shown in Table 17.

For the significant relationships shown in Tables 12-17,Table 18 summarizes the design features and quality categories (in parenthesis) with the comparing MPSE level listed in the column header and the reference MPSE level in the row header. For example, A8 (browse PA goals; A/O) is listed under the column H (high) and the row M (medium) because high-MPSE participants, compared with medium-MPSE participants, are more likely to categorize this design feature into *attractive* or *one-dimensional*. This table indicates that high-MPSE participants more likely categorize design features into the *valued* category than the other two groups, as *6* design features were associated with relative likelihoods of *valued* versus *indifferent* by the high-MPSE group, whereas only *1* design feature was associated by the low group and *none* by the medium group in contrast. The finding also revealed that the medium group more likely categorizes design features into *must-be*, as all of the six relationships categorized into the *must-be* category were found when medium-MPSE was compared with the other two MPSE levels.

The third research question can be answered with integrating the analysis results from the Kano analysis and the multinomial regression. The two predictors could influence customers’ quality perception to a varying degree. As the Kano analysis revealed an increasing trend in the number of positive design features as PA decreases, PA might have a negative influence. However, the multinomial regression suggested changes of PA only influenced quality categorization in A3. Since A3 was not the winning category as indicated in Table 11, the effect by PA level could be very small. In contrast, MPSE was tested as significant in 14 regression models, of which 6 were the dominant categories according to Table 18. The reasoning suggests that the influence of MPSE on quality perception should be stronger than that of PA.

**Table 15:** Parameter estimates of change of mobile phone self-efficacy (MPSE) from medium to high (*valued* [*attractive+one-dimensional*] over *indifferent*).

Design feature	Mobile phone self-efficacy (H vs L)				95% CI for odds ratio (lower bound-upper bound)
	*b*	Wald	Significance	Odds ratio	
A3	2.622	5.6	.02	13.77	1.57-120.51
A6	0.930	2.2	.14	2.54	0.75-8.61
A8	1.540	5.8	.02	4.66	1.33-16.37
A10	1.522	5.0	.03	4.58	1.21-17.39
A13	1.673	6.4	.01	5.33	1.47-19.37
A18	0.788	1.8	.18	2.20	0.70-6.91
A22	-0.120	0.0	.84	0.89	0.28-2.86
A25	0.511	0.7	.39	1.67	0.52-5.38
A26	1.154	3.4	.07	3.17	0.92-10.88
A29	1.112	3.4	.06	3.04	0.94-9.83
A35	20.051	719.9	.00	5.11E-8	1.18E-8 to 2.21E-9
A41	1.573	4.9	.03	4.82	1.19-19.49
A45	0.431	0.5	.47	1.54	0.48-4.92
A48	0.692	1.2	.27	2.00	0.59-6.83

**Table 16:** Parameter estimates of change of mobile phone self-efficacy (MPSE) from low to high (*valued* [*attractive* + *one-dimensional*] over *indifferent*).

Design feature	Mobile phone self-efficacy (H vs L)				95% CI for odds ratio (lower bound-upper bound)
	*b*	Wald	Significance	Odds ratio	
A3	0.948	2.1	.15	2.58	0.71-9.43
A6	0.698	1.7	.20	2.01	0.70-5.78
A8	0.309	0.3	.56	1.36	0.48-3.84
A10	0.198	0.1	.73	1.22	0.40-3.73
A13	0.887	2.6	.11	2.43	0.83-7.13
A18	0.189	0.1	.72	1.21	0.43-3.38
A22	0.569	1.2	.28	1.77	0.63-4.97
A25	1.048	3.7	.05	2.85	0.98-8.26
A26	1.237	4.8	.03	3.44	1.15-10.35
A29	0.908	2.8	.10	2.48	0.85-7.25
A35	1.010	1.8	.18	2.75	0.64-11.88
A41	0.398	0.6	.46	1.49	0.52-4.24
A45	0.455	0.7	.40	1.58	0.55-4.52
A48	0.213	0.2	.69	1.24	0.43-3.57

**Table 17:** Parameter estimates of change of Mobile phone self-efficacy (MPSE) from low to medium (*valued* [*attractive* + *one-dimensional*] over *indifferent*).

Design feature	Mobile phone self-efficacy (H vs L)				95% CI for odds ratio (lower bound-upper bound)	
	*b*	Wald	Significance	Odds ratio		
A3	1.674	2.2	.14	5.33		0.57-49.82
A6	0.232	0.1	.71	1.26		0.37-4.25
A8	1.231	3.8	.05	3.42		0.99-11.83
A10	1.324	4.0	.047	3.76		1.02-13.85
A13	0.786	1.5	.23	2.20		0.62-7.81
A18	0.599	1.0	.32	1.82		0.56-5.93
A22	−0.689	1.3	.25	0.50		0.16-1.62
A25	−0.537	0.8	.38	0.59		0.18-1.93
A26	−0.083	0.0	.90	0.92		0.27-3.15
A29	0.203	0.1	.72	1.23		0.40-3.79
A35	19.041	N/A^a^	N/A	1.86E-8		1.86E-8 to 1.86E-8
A41	1.176	2.6	.11	3.24		0.77-13.69
A45	−0.024	0.0	.97	0.98		0.298-3.194
A48	0.480	0.5	.46	1.62		0.453-5.764

^a^N/A: not applicable.

**Table 18:** The quality categories of design features with significant mobile phone self-efficacy (MPSE) coefficients.

	H^a^	M^a^	L^a^
H^b^		A22: contingent rewards with grading or shaping (M)^c^	
M^b^	A3: social approval (A/O) A8: browse PA^d^ goals (A/O) A10: remind to record PA (A/O) A13: browse health goals (A/O)^e^ A35: PA fulfilled as a contract (A/O) A41: talk to role models (A/O)		A10: remind to record PA (A/O)
L^b^	A26: comparing actual PA with PA goal (A/O)^e^	A6: set PA goals (M) A22: contingent rewards with grading or shaping (M)^c^ A25: PA history (M)^c^ A26: comparing actual PA with PA goal (M)^c^ A45: prompt mental imagery (M)	

^a^Comparing level represented by a dummy variable.

^b^Reference level.

^c^This category also listed as a winning category in the Kano analysis on the medium-PA participants.

^d^PA: physical activity.

^e^This category also listed as a winning category in the Kano analysis on the high-PA participants.

## Discussion

### Principal Findings

The Kano analyses provide evidence with which to answer the research questions. Overall, BCT-based exercise-promoting features that can attract middle-agers are limited. The analysis on the total sample revealed that visual demonstration of exercise instructions (A30) may be the only *attractive* design feature, whereas the other 51 design features are perceived as *indifferent*. This result is not surprising, as studies have reported physical inactivity in the middle-aged population [57,58], explaining a potential lack of motivation to use these persuasive features. Our Kano analyses also suggest 6 positive design features for mobile phone users with middle-PA, 21 for low-PA users, whereas also recommending 14 for high-MPSE, 6 for medium-MPSE, and 1 for low-MPSE users. The second research problem is answered by comparing the Kano analysis results to the prevalence numbers proposed in [34]. The analysis suggests that the implementation of demanded features could be low, as the average prevalence of demanded design features is 20% (4.3/21).

The third research question is answered with the findings obtained by the Kano analysis and the multinomial regression analyses. We found that both PA and MPSE could influence customers’ quality perception, whereas the magnitude of effect is larger for MPSE, because MPSE effects quality perception of more design features than PA.

### Implications for Design Features

Customization has been proposed as important to mHealth apps [12,59], assuming that users with different characteristics are associated with different needs. Our finding is consistent with this assumption, as the quality perception differs across the levels of PA and MPSE. Accordingly, apps should measure users’ PA and MPSE for customization settings. However, inappropriate customization (eg, too many or incorrect features) will overload users with cognitive complexity which increases errors and reduces operational efficiency. Besides, adding more choices and options to a single user interface will create uncertainty and induce distraction, finally leading to negative experience [60]. Therefore, customization requires knowledge regarding the right functions delivered to the right users who need them. This study contributes with the design recommendations grounded in the Kano study with which exercise-promotion apps can adapt the function provision and user interface to user needs and enhance the positive user experience.

Health care experts and app designers are often challenged with the question of what functions to offer, as the number of exercise-promotion apps is rapidly increasing [61]. The *attractive* features offered in our Kano study can shed light on the answer, as focusing on attractive quality attributes will outperform only providing expected quality attributes in maintaining strategic advantage [62]. Besides, implementing attractive designs may incur no risk, as low performance in such designs will not increase customer dissatisfaction as defined in the Kano model. One function to build for *all* middle-agers is visual demonstration (A30), as visual demonstration is the sole attractive design to the total sample. Even though the prevalence of this function has been proposed as high as 47% [18], app designers are still encouraged to delve into more varieties of this feature (eg, exercise demonstration with virtual reality [63]). Besides, our analysis also reveals 4 features (ie, A12, A24, A29, and A52) which should be provided to low-PA middle-agers, whereas A11 should be delivered to medium-PA users. This suggestion echoes the importance of behavioral monitoring in PA promotion [34], as A11 (automatically record PA), A12 (set health goals), and A52 (reminding to PA) relates to behavioral facilitation. Furthermore, as motivational interviewing and self-talk were not present in any app analyzed in [33], offering A24 (reminding past success in exercise) would create unique value in the market.

For three design features in demand but with little or no supply (ie, A21, A22, and A23), we propose design guidelines based on existing findings in the literature. For A23 (prompt generalization of exercise), we suggest app designers including indoor maps (eg, Google Indoor Maps) and remind users with location-based messages to encourage stair use. For example, when users are very close to a stair, messages invoking heuristic processing (eg, use the stair) should be used, whereas when a stair is placed at some distance allowing systematic processing, messages should be designed to induce systematic processing such as “will you take the stair? [64]” To perform well in A21 (contingent reward for exercise practice) and A22 (contingent rewards with grading), an app should collect information regarding user practicing PA and offer users with rewards contingent on user behavior. As existing studies have proposed diversified reward structures [65,66], app designers should implement the reward structures and test their effectiveness in a natural setting. Apps can exercise persuasive appeals to induce intrinsic motivation so that users can understand the importance of exercise. This requirement is also reflected in our finding, as A1 (general consequences of exercise) and A2 (customized consequences of exercise) are categorized as *must-be* by low-PA participants. As with extrinsic motivation, app designers can consider to cooperate with advertising agencies and provide users with economic incentives (eg, The AIR MILES incentives [67]).

Although comparing needs against features supplied, social comparison and social support were surprisingly rated as *indifferent*, even in the high-MPSE subsample. This finding seems contrary to the popularity of social networking functions in exercise-promotion apps for the younger population. As most of the participants used social networking apps (eg, LINE and Facebook), we therefore assumed that they should be aware of social networking apps and own experiences in basic functions, which suggests that middle-aged adults did not intend to receive social support or conduct social comparison via exercise-promotion apps. Possible explanations are as follows: for those middle-agers who overlap PA with social life, they may have formed own styles to interact with exercise partners. On the other hand, a habit of separating exercise from social life also weakens the need for sociability of exercise-promotion apps. Accordingly, this study suggests that app developers should consider keeping social networking functionality to a minimum extent, and allowing users to disable functions or hide related widgets in the interface from being seen. Another recommendation is to connect with existing social networking apps (eg, LINE and Facebook) in order to minimize the cognitive load in learning new apps.

### Comparison With Prior Work

The formative study was reported by Rabin and Bock [21], who proposed that mobile phone users had a number of specific preferences with regard to PA. This study is in line with [21] in increasing understanding on how mobile phone users perceive app features promoting PA. Uniquely, this study is grounded with the BCTs proposed by Michie et al [22] and targets the cohort of middle-aged adults, therefore offering more insights on their appraisals on mobile phone features promoting PA. To our knowledge, this work is also the first to address middle-agers’ quality perception toward design features of exercise-promotion apps. With our empirical findings, this study offers strategic recommendations for app developers to create value with attractive features that can induce positive emotion.

Existing works have proposed that technology self-efficacy can influence perceived usefulness on computing devices in health care [68]. This study is in concordance with [68] in understanding the effects of technology self-efficacy on perception toward technology use. Uniquely, our finding contributes to current literature in the finding that MPSE influences quality categorization. In particular, high self-efficacy seems to make features look more *attractive*, whereas medium self-efficacy only considers something she or he must have. This finding not only contributes to the self-efficacy studies with new evidence, but also informs practitioners of the importance of increasing user’s confidence.

### Limitations

The limitations of this study include the small sample size. The findings are also limited in that the participants were not based on probability sampling. As we interpreted the Kano analysis results in an aggressive approach, the effect of *indifferent* perception was therefore weakened through the analysis. A larger sample should be used to alleviate these issues in future study design.

To avoid participant fatigue, we created the questionnaire based on the 40 BCTs proposed by Michie et al [22]. Future studies are recommended to adopt the 93 techniques in the BCT taxonomy (v1) to generate more design features. This study is also limited due to its cross-sectional design. With randomized controlled trials, a study can more easily attribute any observed effect to the treatments being compared, from which strong evidence can be derived [69].

### Conclusions and Future Research

Patient-centered care (PCC) advocates that patient needs and preferences should be respected [70]. Following the PCC principle, this study fills the research gap by offering the design recommendations of exercise-promotion mobile phone apps for middle-agers. Visual demonstration is the sole feature that should be implemented for middle-agers, whereas design features customized for middle-aged adults of different characteristics are also provided. By comparing the needs in our findings to the current supply of app features, attractive design features are suggested to enhance strategic advantage of app developers. In addition to these recommended app features, MPSE is identified as a dominant factor inducing attractive and one-dimensional quality perception, where quality categorization by high-MPSE participants mostly (ie, 12/14) falls into *one-dimensional*, whereas all (6/6) of the non- *indifferent* categories by medium MPSE participants are *must-be*. The results by the multinomial regression analysis also indicated a similar pattern. Although current literature have proposed that self-efficacy influences perceived usefulness and perceived ease of use [68], the relationship between technology-use self-efficacy and quality categorization may remain an open question. This calls for future research to explore the underlying mechanisms behind the findings.

## Appendix

Multimedia Appendix 1 The scoring protocol of IPAQ-Taiwan. IPAQ: International Physical Activity Questionnaire.

Multimedia Appendix 2 Kano analysis results.

Multimedia Appendix 3 Model fitting information and the results of likelihood ratio tests.

## Abbreviations

BCT: behavior change technique
BMI: body mass index
CALO-RE: Coventry, Aberdeen, and London—Refined
CGMH: Chang Gung Memorial Hospital
CPU: central processing unit
IPAQ: International Physical Activity Questionnaire
IPAQ-LF: International Physical Activity Questionnaire-long form
IPAQ-SF: International Physical Activity Questionnaire-short form
IRB: Institutional Review Board
IT: information technology
OR: odds ratio
PA: physical activity
PCC: patient-centered care
SMS: short message service
UTAUT: unified theory of acceptance and use of technology
