# Supplementary material for: What to Build for Middle-Agers to Come? Attractive and Necessary Functions of Exercise-Promotion Mobile Phone Apps: A Cross-Sectional Study
Source: JMIR Mhealth Uhealth. 2017 May 25;5(5):e65. doi: 10.2196/mhealth.6233 (PMC5465381; doi:10.2196/mhealth.6233)
Supplement: Multimedia Appendix 1 [file mhealth_v5i5e65_app1.pdf]

Appendix 1: The scoring protocol of IPAQ-Taiwan

The long form of IPAQ-Taiwan asks in detail about walking, moderate-intensity and vigorous-intensity physical activity in each of the four domains. This long form version of IPAQ-Taiwan modifies the original IPAQ-LF (long form) version with cultural adaptation and follows the same scoring protocol, which is described in this appendix.

Continuous Score

Data collected with the IPAQ-Taiwan long form can be reported as a continuous measure and reported as median MET-minutes. Median values and interquartile ranges can be computed for walking (W), moderate-intensity activities (M), and vigorous-intensity activities (V) within each domain using the formulas below. Total scores may also be calculated for walking (W), moderate-intensity activities (M), and vigorous-intensity activities (V); for each domain (work, transport, domestic and garden, and leisure) and for an overall grand total.

MET Values and Formula for Computation of MET-minutes

<u>Work Domain</u>

Walking MET-minutes/week at work = 3.3 * walking minutes * walking days at work

Moderate MET-minutes/week at work= 4.0 * moderate-intensity activity minutes * moderate-intensity days at work

Vigorous MET-minutes/week at work= 8.0 * vigorous-intensity activity minutes * vigorous-intensity days at work

Total Work MET-minutes/week =sum of Walking + Moderate + Vigorous MET-minutes/week scores at work.

<u>Active Transportation Domain</u>

Walking MET-minutes/week for transport = 3.3 * walking minutes * walking days for transportation

Cycle MET-minutes/week for transport= 6.0 * cycling minutes * cycle days for transportation

Total Transport MET-minutes/week = sum of Walking + Cycling MET-minutes/week scores for transportation.

Domestic and Garden [Yard Work] Domain

Vigorous MET-minutes/week yard chores= 5.5 * vigorous-intensity activity minutes * vigorous-intensity days doing yard work (**Note**: the MET value of 5.5 indicates that vigorous garden/yard work should be considered a moderate-intensity activity for scoring and computing total moderate intensity activities.)

Moderate MET-minutes/week yard chores= 4.0 * moderate-intensity activity minutes * moderate-intensity days doing yard work

Moderate MET-minutes/week inside chores= 3.0* moderate-intensity activity minutes * moderate-intensity days doing inside chores.

Total Domestic and Garden MET-minutes/week =sum of Vigorous yard + Moderate yard + Moderate inside chores MET-minutes/week scores.

Leisure-Time Domain

Walking MET-minutes/week leisure = 3.3 * walking minutes * walking days in leisure

Moderate MET-minutes/week leisure = 4.0 * moderate-intensity activity minutes * moderate-intensity days in leisure

Vigorous MET-minutes/week leisure = 8.0 * vigorous-intensity activity minutes * vigorous-intensity days in leisure

Total Leisure-Time MET-minutes/week = sum of Walking + Moderate + Vigorous MET-minutes/week scores in leisure.

Total Scores for all Walking, Moderate and Vigorous Physical Activities

Total Walking MET-minutes/week = Walking MET-minutes/week (at Work + for Transport + in Leisure)

Total Moderate MET-minutes/week total = Moderate MET-minutes/week (at Work + Yard chores + inside chores + in Leisure time) + Cycling Met-minutes/week for Transport + Vigorous Yard chores MET-minutes/week

Total Vigorous MET-minutes/week = Vigorous MET-minutes/week (at Work + in Leisure)

Note: Cycling MET value and Vigorous garden/yard work MET value fall within the coding range of moderate-intensity activities.

Total Physical Activity Scores

An overall total physical activity MET-minutes/week score can be computed as:

Total physical activity MET-minutes/week = sum of Total (Walking + Moderate + Vigorous) MET-minutes/week scores.

This is equivalent to computing:

Total physical activity MET-minutes/week = sum of Total Work + Total Transport + Total Domestic and Garden + Total Leisure-Time MET-minutes/week scores.

As there are no established thresholds for presenting MET-minutes, the IPAQ Research Committee proposes that these data are reported as comparisons of median values and interquartile ranges for different populations.

Categorical Score

As noted earlier, regular participation is a key concept included in current public health guidelines for physical activity. Therefore, both the total volume and the number of day/sessions are included in the IPAQ analysis algorithms. There are three levels of physical activity proposed to classify populations – ‘low’, ’moderate’, and ‘high’.

Category 1 Low

This is the lowest level of physical activity. Those individuals who not meet criteria for Categories 2 or 3 are considered ‘low’.

Category 2 Moderate

The pattern of activity to be classified as ‘moderate’ is either of the following criteria:

3 or more days of vigorous-intensity activity of at least 20 minutes per day

OR

5 or more days of moderate-intensity activity and/or walking of at least 30 minutes per day

OR

5 or more days of any combination of walking, moderate-intensity or vigorous-intensity activities achieving a minimum Total physical activity of at least 600 MET-minutes/week.

Individuals meeting at least one of the above criteria would be defined as accumulating a moderate level of activity.

Category 3 High

A separate category labelled ‘high’ can be computed to describe higher levels of participation. The two criteria for classification as ‘high’ are:

vigorous-intensity activity on at least 3 days achieving a minimum Total physical activity of at least 1500 MET-minutes/week

OR

7 or more days of any combination of walking, moderate-intensity or vigorous-intensity activities achieving a minimum Total physical activity of at least 3000 MET-minutes/week.

Reference from: IPAQ Research Committee. (2005). Guidelines for data processing and analysis of the International Physical Activity Questionnaire (IPAQ)–short and long forms.
