# Supplementary material for: What to Build for Middle-Agers to Come? Attractive and Necessary Functions of Exercise-Promotion Mobile Phone Apps: A Cross-Sectional Study
Source: JMIR Mhealth Uhealth. 2017 May 25;5(5):e65. doi: 10.2196/mhealth.6233 (PMC5465381; doi:10.2196/mhealth.6233)
Supplement: Multimedia Appendix 2 [file mhealth_v5i5e65_app2.pdf]

Appendix 2: KANO analysis results.

This appendix details the analysis results with the KANO method. Specifically, The KANO method was conducted on participants with different characteristics and the analysis results are shown in Table 1 (the total sample), Table 2 (the high-PA sub-sample), Table3 (the medium-PA sub-sample), Table 4 (the low-PA sub-sample), Table 5 (the high-SSE sub-sample), Table 6 (the medium-SSE sub-sample), and Table 7 (the low-SSE sub-sample). The notation $X(C_1, C_2, \ldots, C_n)$ in the tables indicates a design feature had close proportions in the categories of C_i, $1 \leq i \leq n$.

Table 1. Categorizing design features according to total sample (n=103)

Design feature	Frequency of design feature						Category strength (%)	Total strength (%)	Classification results
	A	M	O	I	R	Q			
A1	9	30	9	52	2	1	21	47	I
A2	12	25	17	48	0	1	23	52	I
A3	12	9	4	69	7	2	55	24	I
A4	8	11	3	76	4	1	63	21	I
A5	10	20	18	52	1	2	31	47	I
A6	13	22	18	48	0	2	25	52	I
A7	14	21	21	42	4	1	20	54	I
A8	21	20	15	45	0	2	23	54	I
A9	16	26	12	48	0	1	21	52	I
A10	17	28	17	39	0	2	11	60	I
A11	19	19	14	50	0	1	30	51	I
A12	13	21	20	47	1	1	25	52	I
A13	13	24	21	43	0	2	18	56	I
A14	14	18	25	42	1	3	17	55	I
A15	13	25	21	41	0	3	16	57	I
A16	17	14	16	55	0	1	37	46	I
A17	18	15	16	53	0	1	34	48	I
A18	21	8	11	60	2	1	38	39	I
A19	17	9	13	62	1	1	44	38	I
A20	21	18	17	44	1	2	22	54	I
A21	17	21	21	40	1	3	18	57	I
A22	17	19	26	39	1	1	13	60	I
A23	17	18	27	37	2	2	10	60	I

A24	21	16	16	47	1	2	25	52	I
A25	16	22	21	42	0	2	19	57	I
A26	12	24	23	42	0	2	18	57	I
A27	14	13	8	65	1	2	50	34	I
A28	24	16	14	46	1	2	21	52	I
A29	21	17	26	36	1	2	10	62	I
A30	28	15	23	33	1	3	5	64	X(I, A)
A31	21	11	15	53	0	3	31	46	I
A32	26	11	15	48	1	2	21	51	I
A33	23	9	13	55	1	2	31	44	I
A34	23	10	14	53	1	2	29	46	I
A35	6	5	4	68	16	4	51	15	I
A36	13	8	14	64	2	2	49	34	I
A37	14	7	9	68	2	3	52	29	I
A38	18	7	7	64	5	2	45	31	I
A39	23	8	7	60	3	2	36	37	I
A40	18	6	6	68	3	2	49	29	I
A41	17	4	7	67	5	3	49	27	I
A42	12	6	11	70	3	1	56	28	I
A43	12	11	12	59	6	3	46	34	I
A44	21	14	12	54	1	1	32	46	I
A45	16	14	13	57	1	2	40	42	I
A46	21	12	13	54	1	2	32	45	I
A47	19	11	13	57	2	1	37	42	I
A48	13	9	12	67	1	1	52	33	I
A49	18	10	14	60	0	1	41	41	I
A50	22	11	12	56	0	2	33	44	I
A51	15	9	8	67	2	2	51	31	I
A52	21	21	20	40	0	1	18	60	I

Table 2. Categorizing design features according to high level of physical activity (n=62)

Design feature	Frequency of design feature						Category strength (%)	Total strength (%)	Classification results
	A	M	O	I	R	Q			
A1	8	14	4	35	1	0	34	42	I
A2	8	15	7	32	0	0	27	48	I
A3	9	5	4	40	4	0	50	29	I
A4	4	7	3	46	2	0	63	23	I
A5	5	10	8	37	1	1	44	37	I
A6	9	12	9	31	0	1	31	48	I
A7	8	13	10	29	2	0	26	50	I
A8	11	9	11	30	0	1	31	50	I
A9	8	14	8	32	0	0	29	48	I
A10	10	15	8	29	0	0	23	53	I
A11	8	12	9	33	0	0	34	47	I
A12	5	12	13	32	0	0	31	48	I
A13	7	11	14	29	0	1	24	52	I
A14	8	9	14	30	0	1	26	50	I
A15	7	14	12	28	0	1	23	53	I
A16	9	7	10	36	0	0	42	42	I
A17	10	9	9	34	0	0	39	45	I
A18	10	4	6	40	2	0	48	32	I
A19	8	5	6	42	1	0	55	31	I
A20	13	8	8	31	1	1	29	47	I
A21	10	10	10	30	1	1	32	48	I
A22	8	9	16	28	1	0	19	53	I
A23	11	9	14	26	1	1	19	55	I
A24	12	8	8	33	0	1	34	45	I
A25	10	10	12	29	0	1	27	52	I
A26	7	11	13	30	0	1	27	50	I
A27	10	8	3	39	1	1	47	34	I
A28	15	10	7	28	1	1	21	52	I
A29	10	9	15	26	1	1	18	55	I
A30	16	7	13	23	1	2	11	58	I
A31	12	6	8	34	0	2	36	42	I

A32	15	6	7	32	1	1	27	45	I
A33	15	6	6	34	0	1	31	44	I
A34	13	7	8	32	1	1	31	45	I
A35	3	4	2	40	10	3	48	15	I
A36	7	6	8	38	2	1	48	34	I
A37	8	5	4	43	1	1	57	27	I
A38	10	5	4	38	4	1	45	31	I
A39	12	5	3	39	2	1	44	32	I
A40	12	4	2	40	3	1	45	29	I
A41	9	3	4	41	3	2	52	26	I
A42	5	5	7	43	2	0	58	27	I
A43	7	7	7	35	5	1	45	34	I
A44	11	9	7	34	1	0	37	44	I
A45	8	9	7	37	1	0	45	39	I
A46	12	9	6	34	1	0	36	44	I
A47	11	9	7	33	2	0	36	44	I
A48	7	7	6	41	1	0	55	32	I
A49	11	8	6	37	0	0	42	40	I
A50	12	9	5	35	0	1	37	42	I
A51	9	7	5	38	2	1	47	34	I
A52	11	12	12	27	0	0	24	57	I

Table 3. Categorizing design features according to medium level of physical activity (n=18)

Design feature	Frequency of design feature						Category strength (%)	Total strength (%)	Classification results
	A	M	O	I	R	Q			
A1	0	5	3	9	1	0	22	44	I
A2	2	2	6	8	0	0	11	56	I
A3	0	3	0	14	1	0	61	17	I
A4	0	2	0	14	2	0	67	11	I
A5	4	3	5	6	0	0	6	67	X(I,O)
A6	2	3	4	9	0	0	28	50	I
A7	3	2	4	8	1	0	22	50	I
A8	6	4	0	8	0	0	11	56	I
A9	5	5	0	8	0	0	17	56	I
A10	4	7	2	5	0	0	11	72	M
A11	6	5	1	6	0	0	0	67	X(A,I)
A12	3	3	2	10	0	0	39	44	I
A13	3	5	2	8	0	0	17	56	I
A14	3	3	3	9	0	0	33	50	I
A15	2	4	2	10	0	0	33	44	I
A16	4	4	1	9	0	0	28	50	I
A17	3	2	2	11	0	0	44	39	I
A18	3	2	2	11	0	0	44	39	I
A19	3	2	3	10	0	0	39	44	I
A20	4	5	3	6	0	0	6	67	X(I,M)
A21	3	4	4	7	0	0	17	61	I
A22	4	2	4	8	0	0	22	56	I
A23	2	3	6	7	0	0	6	61	X(I, O)
A24	3	2	4	8	1	0	22	50	I
A25	2	4	4	8	0	0	22	56	I
A26	1	4	5	8	0	0	17	56	I
A27	1	2	3	12	0	0	50	33	I
A28	3	2	4	9	0	0	28	50	I
A29	4	1	6	7	0	0	6	61	X(I, O)
A30	5	1	5	7	0	0	11	61	I
A31	4	2	3	9	0	0	28	50	I
A32	5	2	3	8	0	0	17	56	I
A33	4	2	3	9	0	0	28	50	I

A34	4	2	3	9	0	0	28	50	I
A35	1	0	1	14	2	0	67	11	I
A36	2	2	3	11	0	0	44	39	I
A37	3	2	2	10	1	0	39	39	I
A38	2	2	2	12	0	0	56	33	I
A39	3	1	3	11	0	0	44	39	I
A40	1	1	3	13	0	0	56	28	I
A41	3	1	2	11	1	0	44	33	I
A42	2	1	2	13	0	0	61	28	I
A43	3	2	3	10	0	0	39	44	I
A44	3	2	3	10	0	0	39	44	I
A45	3	3	3	9	0	0	33	50	I
A46	3	2	3	10	0	0	39	44	I
A47	3	1	3	11	0	0	44	39	I
A48	2	1	3	12	0	0	50	33	I
A49	3	1	4	10	0	0	33	44	I
A50	5	1	3	9	0	0	22	50	I
A51	3	1	2	12	0	0	50	33	I
A52	4	2	5	7	0	0	11	61	I

X(C1, C2): indicates an attribute had close proportions in two categories of C1 and C2.

Table 4. Categorizing design features according to low level of physical activity (n=22)

Design feature	Frequency of design feature						Category strength (%)	Total strength (%)	Classification results
	A	M	O	I	R	Q			
A1	1	11	2	8	0	0	14	64	M
A2	1	8	4	8	0	1	0	59	M
A3	2	1	0	15	2	2	59	14	I
A4	4	2	0	16	0	0	55	27	I
A5	1	7	5	9	0	0	9	59	I
A6	2	7	5	8	0	0	5	64	X(I,M)
A7	3	6	7	5	1	0	5	73	X(O,M)
A8	4	7	4	7	0	0	0	68	X(M,I)
A9	3	7	4	8	0	0	5	64	X(I,M)
A10	3	6	7	5	0	1	5	73	X(O,M)
A11	5	2	4	11	0	0	27	50	I
A12	5	6	5	5	1	0	5	73	X(M,A,O,I)
A13	3	8	5	6	0	0	9	73	M
A14	3	6	8	3	1	1	9	77	O
A15	4	7	7	3	0	1	0	82	X(M,O)
A16	4	3	5	10	0	0	23	55	I
A17	5	4	5	8	0	0	14	64	I
A18	8	2	3	9	0	0	5	59	I
A19	6	2	4	10	0	0	18	55	I
A20	4	5	6	7	0	0	5	68	X(I,O)
A21	4	7	7	3	0	1	0	82	X(M,O)
A22	5	8	6	3	0	0	9	86	M
A23	4	6	7	4	1	0	5	77	X(O,M)
A24	6	6	4	6	0	0	0	73	X(A,M,I)
A25	4	8	5	5	0	0	14	77	M
A26	4	9	5	4	0	0	18	82	M
A27	3	3	2	14	0	0	50	36	I
A28	6	4	3	9	0	0	14	59	I
A29	7	7	5	3	0	0	0	86	X(A,M)
A30	7	7	5	3	0	0	0	86	X(A,M)
A31	5	3	4	10	0	0	23	55	I
A32	6	3	5	8	0	0	9	64	I
A33	4	1	4	12	1	0	36	41	I

A34	6	1	3	12	0	0	27	45	I
A35	2	1	1	14	4	0	45	18	I
A36	4	0	3	15	0	0	50	32	I
A37	3	0	3	15	0	1	55	27	I
A38	6	0	1	14	1	0	36	32	I
A39	8	2	1	10	1	0	9	50	I
A40	5	1	1	15	0	0	45	32	I
A41	5	0	1	15	1	0	45	27	I
A42	5	0	2	14	1	0	41	32	I
A43	2	2	2	14	1	1	55	27	I
A44	7	3	2	10	0	0	14	55	I
A45	5	2	3	11	0	1	27	45	I
A46	6	1	4	10	0	1	18	50	I
A47	5	1	3	13	0	0	36	41	I
A48	4	1	3	14	0	0	45	36	I
A49	4	1	4	13	0	0	41	41	I
A50	5	1	4	12	0	0	32	45	I
A51	3	1	1	17	0	0	64	23	I
A52	6	7	3	6	0	0	5	73	X(M,A,I)

Table 5. Categorizing design features according to high self-efficacy (n=35)

Design feature	Frequency of design feature						Category strength (%)	Total strength (%)	Classification results
	A	M	O	I	R	Q			
A1	2	12	4	15	1	1	9	51	I
A2	6	10	7	11	0	1	3	66	X(I,M)
A3	7	4	3	17	3	1	29	40	I
A4	3	4	1	24	2	1	57	23	I
A5	5	8	6	14	0	2	17	54	I
A6	6	7	8	13	0	1	14	60	I
A7	5	7	9	12	1	1	9	60	I
A8	8	6	8	12	0	1	11	63	I
A9	5	9	7	13	0	1	11	60	I
A10	6	10	8	10	0	1	0	69	X(M,I)
A11	5	8	5	16	0	1	23	51	I
A12	6	5	11	12	0	1	3	63	X(I,O)
A13	7	7	10	9	0	2	3	69	X(O,I)
A14	6	6	11	10	0	2	3	66	X(O,I)
A15	5	8	10	10	0	2	0	66	X(O,I)
A16	6	4	10	14	0	1	11	57	I
A17	7	4	10	13	0	1	9	60	I
A18	7	0	7	19	1	1	34	40	I
A19	6	3	7	18	0	1	31	46	I
A20	9	4	7	13	0	2	11	57	I
A21	7	5	10	11	0	2	3	63	X(I,O)
A22	4	3	14	12	1	1	6	60	X(O,I)
A23	6	3	14	10	0	2	11	66	O
A24	9	3	8	13	0	2	11	57	I
A25	6	4	12	11	0	2	3	63	X(O,I)
A26	5	6	13	9	0	2	11	69	O
A27	7	4	4	18	0	2	31	43	I
A28	10	4	5	13	1	2	9	54	I
A29	7	3	15	8	0	2	20	71	O
A30	9	3	13	7	1	2	11	71	O
A31	8	4	7	13	0	3	14	54	I

A32	10	3	8	12	0	2	6	60	I
A33	7	2	8	16	0	2	23	49	I
A34	7	3	7	15	1	2	23	49	I
A35	4	2	3	17	7	2	29	26	I
A36	5	2	7	18	1	2	31	40	I
A37	4	1	5	22	1	2	49	29	I
A38	9	1	4	19	0	2	29	40	I
A39	9	1	3	19	1	2	29	37	I
A40	9	1	3	19	2	1	29	37	I
A41	8	0	4	19	2	2	31	34	I
A42	6	1	5	21	1	1	43	34	I
A43	5	2	5	19	3	1	40	34	I
A44	7	5	7	14	1	1	20	54	I
A45	6	4	6	17	1	1	31	46	I
A46	8	3	6	16	1	1	23	49	I
A47	7	1	5	21	0	1	40	37	I
A48	6	0	5	22	1	1	46	31	I
A49	9	1	5	19	0	1	29	43	I
A50	9	1	4	19	0	2	29	40	I
A51	6	1	4	21	1	2	43	31	I
A52	6	6	10	12	0	1	6	63	X(I,O)

Table 6. Categorizing design features according to medium self-efficacy (n=32)

Design feature	Frequency of design feature						Category strength (%)	Total strength (%)	Classification results
	A	M	O	I	R	Q			
A1	3	13	1	15	0	0	6	53	I
A2	2	10	2	18	0	0	25	44	I
A3	1	3	0	26	1	1	72	13	I
A4	2	3	1	25	1	0	69	19	I
A5	1	9	4	17	1	0	25	44	I
A6	3	12	3	14	0	0	6	56	I
A7	3	11	4	13	1	0	6	56	I
A8	3	10	2	16	0	1	19	47	I
A9	3	11	2	16	0	0	16	50	I
A10	2	12	3	14	0	1	6	53	I
A11	4	7	5	16	0	0	28	50	I
A12	2	10	4	15	1	0	16	50	I
A13	1	12	4	15	0	0	9	53	I
A14	3	6	7	15	1	0	25	50	I
A15	3	12	4	12	0	1	0	59	M
A16	6	5	2	19	0	0	41	41	I
A17	5	6	2	19	0	0	41	41	I
A18	6	5	1	19	1	0	41	38	I
A19	7	4	2	18	1	0	34	41	I
A20	5	10	4	13	0	0	9	59	I
A21	5	10	3	12	1	1	6	56	I
A22	7	12	5	8	0	0	13	75	M
A23	6	10	4	10	2	0	0	63	X(M,I)
A24	5	10	3	14	0	0	13	56	I
A25	6	13	3	10	0	0	9	69	M
A26	4	13	3	12	0	0	3	63	X(M,I)
A27	3	7	1	20	1	0	41	34	I
A28	5	8	3	16	0	0	25	50	I
A29	5	10	5	12	0	0	6	63	I
A30	7	8	3	13	0	1	16	56	I
A31	6	5	4	17	0	0	34	47	I
A32	7	5	2	18	0	0	34	44	I
A33	9	5	2	15	1	0	19	50	I

A34	7	5	3	17	0	0	31	47	I
A35	0	2	0	26	3	1	72	6	I
A36	3	5	3	21	0	0	50	34	I
A37	5	5	2	19	0	1	44	38	I
A38	4	4	1	21	2	0	53	28	I
A39	5	5	1	20	1	0	47	34	I
A40	4	4	0	23	1	0	59	25	I
A41	3	3	0	23	2	1	63	19	I
A42	3	4	2	22	1	0	56	28	I
A43	2	6	3	20	0	1	44	34	I
A44	7	7	1	17	0	0	31	47	I
A45	5	9	2	15	0	1	19	50	I
A46	6	6	2	18	0	0	38	44	I
A47	3	7	4	16	2	0	28	44	I
A48	3	7	2	20	0	0	41	38	I
A49	4	7	3	18	0	0	34	44	I
A50	7	6	2	17	0	0	31	47	I
A51	4	5	1	21	1	0	50	31	I
A52	7	12	3	10	0	0	6	69	M

Table 7. Categorizing design features according to low self-efficacy(n=35)

Design feature	Frequency of design feature						Category strength (%)	Total strength (%)	Classification results
	A	M	O	I	R	Q			
A1	4	5	4	21	1	0	46	37	I
A2	4	5	8	18	0	0	29	49	I
A3	4	2	1	25	3	0	60	20	I
A4	3	4	1	26	1	0	63	23	I
A5	4	3	8	20	0	0	34	43	I
A6	4	3	7	20	0	1	37	40	I
A7	6	3	8	16	2	0	23	49	I
A8	10	4	5	16	0	0	17	54	I
A9	8	6	3	18	0	0	29	49	I
A10	9	6	6	14	0	0	14	60	I
A11	10	4	4	17	0	0	20	51	I
A12	5	6	5	19	0	0	37	46	I
A13	5	5	7	18	0	0	31	49	I
A14	5	6	7	16	0	1	26	51	I
A15	5	5	7	18	0	0	31	49	I
A16	5	5	4	21	0	0	46	40	I
A17	6	5	4	20	0	0	40	43	I
A18	8	3	3	21	0	0	37	40	I
A19	4	2	4	25	0	0	60	29	I
A20	7	4	6	17	1	0	29	49	I
A21	5	6	8	16	0	0	23	54	I
A22	6	4	7	18	0	0	31	49	I
A23	5	5	9	16	0	0	20	54	I
A24	7	3	5	19	1	0	34	43	I
A25	4	5	6	20	0	0	40	43	I
A26	3	5	7	20	0	0	37	43	I
A27	4	2	3	26	0	0	63	26	I
A28	9	4	6	16	0	0	20	54	I
A29	9	4	6	15	1	0	17	54	I
A30	12	4	7	12	0	0	0	66	X(A,I)
A31	7	2	4	22	0	0	43	37	I
A32	9	3	5	17	1	0	23	49	I
A33	7	2	3	23	0	0	46	34	I

A34	9	2	4	20	0	0	31	43	I
A35	2	1	1	24	6	1	51	11	I
A36	5	1	4	24	1	0	54	29	I
A37	5	1	2	26	1	0	60	23	I
A38	5	2	2	23	3	0	51	26	I
A39	9	2	3	20	1	0	31	40	I
A40	5	1	3	25	0	1	57	26	I
A41	6	1	3	24	1	0	51	29	I
A42	3	1	4	26	1	0	63	23	I
A43	5	3	4	19	3	1	40	34	I
A44	7	2	4	22	0	0	43	37	I
A45	5	1	5	24	0	0	54	31	I
A46	7	3	5	19	0	1	34	43	I
A47	9	3	4	19	0	0	29	46	I
A48	4	2	5	24	0	0	54	31	I
A49	5	2	6	22	0	0	46	37	I
A50	6	4	6	19	0	0	37	46	I
A51	5	3	3	24	0	0	54	31	I
A52	8	3	7	17	0	0	26	51	I
