# Supplementary material for: What to Build for Middle-Agers to Come? Attractive and Necessary Functions of Exercise-Promotion Mobile Phone Apps: A Cross-Sectional Study
Source: JMIR Mhealth Uhealth. 2017 May 25;5(5):e65. doi: 10.2196/mhealth.6233 (PMC5465381; doi:10.2196/mhealth.6233)
Supplement: Multimedia Appendix 3 [file mhealth_v5i5e65_app3.pdf]

Appendix 3: Model fitting information and the results of likelihood ratio tests

	Model fitting				Likelihood Ratio Test			
					Smartphone Self-Efficacy		Level of Physical Activity	
	$\chi^2_{Pearson}$	Sig.	$\chi^2_{Deviance}$	Sig.	χ^2	Sig.	χ^2	Sig.
A1	10.090 (df=8)	.26	11.751 (df=8)	.16	6.033 (df=4)	.20	4.428 (df=4)	.35
A2	9.875 (df=8)	.27	11.667 (df=8)	.17	7.881 (df=4)	.10	4.081 (df=4)	.40
A3	3.566 (df=8)	.89	5.088 (df=8)	.75	10.172 (df=4)	.04	10.625(df=4)	.03
A4	6.706 (df=8)	.57	8.271 (df=8)	.41	0.494 (df=4)	.97	5.501 (df=4)	.24
A5	8.375 (df=8)	.40	8.643 (df=8)	.37	6.568 (df=4)	.16	7.567 (df=4)	.11
A6	9.213 (df=8)	.33	12.027 (df=8)	.15	10.431 (df=4)	.03	1.244 (df=4)	.87
A7	10.946 (df=8)	.21	13.891 (df=8)	.09	8.164 (df=4)	.09	4.232 (df=4)	.38
A8	5.980 (df=8)	.65	6.253 (df=8)	.62	10.605 (df=4)	.03	3.349 (df=4)	.50
A9	7.866 (df=8)	.45	8.290 (df=8)	.41	5.588 (df=4)	.23	1.460 (df=4)	.83
A10	9.868 (df=8)	.27	11.994 (df=8)	.15	10.418 (df=4)	.03	5.490 (df=4)	.24
A11	3.562 (df=8)	.89	3.772 (df=8)	.88	3.348 (df=4)	.50	5.051 (df=4)	.28
A12	5.487 (df=8)	.70	7.432 (df=8)	.49	8.905 (df=4)	.06	4.405 (df=4)	.35
A13	4.059 (df=8)	.85	4.356 (df=8)	.82	12.168 (df=4)	.02	4.103 (df=4)	.39
A14	4.017 (df=8)	.86	4.527 (df=8)	.81	3.380 (df=4)	.50	5.560 (df=4)	.24
A15	4.730 (df=8)	.79	4.999 (df=8)	.76	7.551 (df=4)	.11	7.523 (df=4)	.11
A16	9.989 (df=8)	.27	11.360 (df=8)	.18	4.766 (df=4)	.31	2.113 (df=4)	.72
A17	11.027 (df=8)	.20	13.712 (df=8)	.09	6.677 (df=4)	.15	3.091 (df=4)	.54
A18	4.482 (df=8)	.81	4.352 (df=8)	.82	9.808 (df=4)	.04	5.615 (df=4)	.23
A19	8.280 (df=8)	.41	8.871 (df=8)	.35	3.042 (df=4)	.55	4.234 (df=4)	.38
A20[a]	13.598 (df=8)	.09	15.569 (df=8)	.049	Excluded from the predictor analysis			
A21	5.722 (df=8)	.68	6.545 (df=8)	.59	5.533 (df=4)	.24	7.682 (df=4)	.10
A22	8.624 (df=8)	.38	10.697 (df=8)	.22	10.980 (df=4)	.03	8.786 (df=4)	.07
A23	6.123 (df=8)	.63	8.263 (df=8)	.41	8.126 (df=4)	.09	3.311 (df=4)	.51
A24	8.322 (df=8)	.40	10.941 (df=8)	.21	9.336 (df=4)	.05	4.624 (df=4)	.33
A25	1.706 (df=8)	.99	1.750 (df=8)	.99	12.758 (df=4)	.01	4.738 (df=4)	.32
A26	7.860 (df=8)	.45	9.076 (df=8)	.34	12.408 (df=4)	.02	6.853 (df=4)	.14
A27	4.945 (df=8)	.76	5.071 (df=8)	.75	7.380 (df=4)	.12	0.046 (df=4)	.99
A28	9.449 (df=8)	.31	11.773 (df=8)	.16	4.529 (df=4)	.34	0.641 (df=4)	.96
A29	4.685 (df=8)	.79	5.885 (df=8)	.66	10.087 (df=4)	.04	9.267 (df=4)	.06
A30	3.534 (df=8)	.90	4.388 (df=8)	.82	8.214 (df=4)	.08	9.377 (df=4)	.05
A31	8.655 (df=8)	.37	9.083 (df=8)	.34	3.617 (df=4)	.46	0.888 (df=4)	.93
A32[a]	14.383 (df=8)	.07	15.852 (df=8)	.045	Excluded from the predictor analysis			
A33	15.154 (df=8)	.06	12.196 (df=8)	.14	4.884 (df=4)	.30	1.490 (df=4)	.83
A34	10.925 (df=8)	.21	7.264 (df=8)	.51	2.694 (df=4)	.61	1.637 (df=4)	.80
A35	2.851 (df=8)	.94	3.185 (df=8)	.92	10.842 (df=4)	.03	2.683 (df=4)	.61
A36	3.313 (df=8)	.91	4.208 (df=8)	.84	6.788 (df=4)	.15	5.435 (df=4)	.25
A37	6.470 (df=8)	.60	6.925 (df=8)	.55	6.695 (df=4)	.15	5.687 (df=4)	.22
A38	2.719 (df=8)	.95	2.964 (df=8)	.94	7.307 (df=4)	.12	4.761 (df=4)	.31
A39	7.736 (df=8)	.46	7.889 (df=8)	.44	5.907 (df=4)	.21	2.722 (df=4)	.61
A40	3.053 (df=8)	.93	4.026 (df=8)	.86	7.596 (df=4)	.11	0.526 (df=4)	.97

A41	3.246 (df=8)	.92	3.314 (df=8)	.91	11.018 (df=4)	.03	3.001 (df=4)	.56
A42	4.019 (df=8)	.86	4.509 (df=8)	.81	6.234 (df=4)	.18	4.731 (df=4)	.32
A43[a]	14.061 (df=8)	.08	17.197 (df=8)	.03	Excluded from the predictor analysis			
A44	12.063 (df=8)	.15	13.154 (df=8)	.11	5.860 (df=4)	.21	1.058 (df=4)	.90
A45	3.066 (df=8)	.93	3.308 (df=8)	.91	11.466 (df=4)	.02	2.782 (df=4)	.60
A46	12.795 (df=8)	.12	11.603 (df=8)	.17	3.783 (df=4)	.44	3.499 (df=4)	.48
A47	12.326 (df=8)	.14	11.480 (df=8)	.18	7.837 (df=4)	.10	3.120 (df=4)	.54
A48	11.999 (df=8)	.15	8.755 (df=8)	.36	13.363 (df=4)	.01	2.542 (df=4)	.64
A49	14.874 (df=8)	.06	11.456 (df=8)	.18	9.021 (df=4)	.06	2.924 (df=4)	.57
A50	9.992 (df=8)	.27	10.091 (df=8)	.26	5.034 (df=4)	.28	4.169 (df=4)	.38
A51	6.258 (df=8)	.62	5.785 (df=8)	.67	4.564 (df=4)	.34	1.963 (df=4)	.74
A52	6.944 (df=8)	.54	7.165 (df=8)	.52	8.235 (df=4)	.08	2.509 (df=4)	.64

[a] Excluded from subsequent analysis due to inappropriate modeling fitting.
